# Can Lung Ultrasound Act as a Diagnosis and Monitoring Tool in Children with Community Acquired Pneumonia? Correlation with Risk Factors, Clinical Indicators and Biologic Results

**DOI:** 10.3390/jcm14155304

**Published:** 2025-07-27

**Authors:** Raluca Isac, Alexandra-Monica Cugerian-Ratiu, Andrada-Mara Micsescu-Olah, Alexandra Daniela Bodescu, Laura-Adelina Vlad, Anca Mirela Zaroniu, Mihai Gafencu, Gabriela Doros

**Affiliations:** 1 Department of Pediatrics, IIIrd Pediatric Clinic, “Victor Babes” University of Medicine and Pharmacy, Eftimie Murgu Square 2, 300041 Timisoara, Romania; isac.raluca@umft.ro (R.I.); mgafencu@umft.ro (M.G.); doros.gabriela@umft.ro (G.D.); 2 Emergency Hospital for Children “Louis Țurcanu”, Iosif Nemoianu Street 2, 300011 Timisoara, Romania; alexandra.cugerian@rezident.umft.ro (A.-M.C.-R.); alexandra.bodescu@rezident.umft.ro (A.D.B.); laura-adelina.vlad@rezident.umft.ro (L.-A.V.); anca.sabadis@student.umft.ro (A.M.Z.)

**Keywords:** lung ultrasound, community-acquired pneumonia, children

## Abstract

**Background:** Community-acquired pneumonia (CAP) is the leading cause of mortality in children from middle- to low-income countries; diagnosing CAP includes clinical evaluation, laboratory testing and pulmonary imaging. Lung ultrasound (LUS) is a sensitive, accessible, non-invasive, non-radiant method for accurately evaluating the lung involvement in acute diseases. Whether LUS findings can be correlated with CAP’s severity or sepsis risk remains debatable. This study aimed to increase the importance of LUS in diagnosing and monitoring CAP. We analyzed 102 children aged 1 month up to 18 years, hospital admitted with CAP. Mean age was 5.71 ± 4.85 years. Underweight was encountered in 44.11% of children, especially below 5 years, while overweight was encountered in 11.36% of older children and adolescents. Patients with CAP presented with fever (79.41%), cough (97.05%), tachypnea (18.62%), respiratory failure symptoms (20.58%), chest pain (12.74%) or poor feeding. Despite the fact that 21.56% had clinically occult CAP and six patients (5.88%) experienced radiologically occult pneumonia, CAP diagnosis was established based on anomalies detected using LUS. **Conclusions:** Detailed clinical examination with abnormal/modified breath sounds and/or tachypnea is suggestive of acute pneumonia. LUS is a sensitive diagnostic tool. A future perspective of including LUS in the diagnosis algorithm of CAP should be taken into consideration.

## 1. Introduction

For decades, pneumonia has been the leading cause of death for children under five years of age living in middle- to low-income countries [1]. Lately, substantial advances have arisen in risk factors’ management, etiology comprehension, early diagnosis, prevention with new vaccine production, new diagnostic techniques, and appropriate and prompt therapy [1,2]. Such developments made a difference in early management of the community-acquired pneumonia (CAP) for the overall outcome, reducing complications and also morbidity of CAP in children [2].

Risk factors for pneumonia include young age, low birth weight, lack of breastfeeding, incomplete vaccination scheme, chronic diseases, pollution, smoke exposure and poverty [3]. Incomplete Vitamin D rickets prophylaxis, Vitamin D deficiency, underweight in children below 5 years, and being overweight in adolescents are known as predisposing factors for CAP in children [3].

Clinical findings in children with CAP include fever, cough, difficult breathing, tachypnea, sometimes mild to severe respiratory failure, chest pain or abdominal discomfort in older children, irritability, and poor feeding in smaller children [4]. Lung auscultation may reveal decreased and/or bronchial breath sounds, rhonchi, crackles, dullness in percussion, cyanosis and/or nasal flaring [4].

Chest X-ray (CXR) is considered the standard assessment for CAP diagnosis, but with both false negative and positive results [5,6]. The need to perform CXRs, considering the risk of irradiation, unneglectable in children, has become a subject of contradiction amongst physicians [5,7]. Cut-off dimensions for identifying pulmonary consolidations on X-ray are considered 1 cm in diameter [8,9,10].

Lung ultrasound (LUS), another possibility for lung imaging, has started to play a much more important role, not only in diagnosis but also in monitoring the evolution of acute pneumonia [7]. LUS is a highly sensitive diagnostic tool in children, which can be performed with a 7–12 linear MHz probe or a 3–5 MHz convex probe, using a great range of sonography devices, with a no-radiation policy. LUS can detect small lesions, starting from 1 mm in diameter, which cannot be perceived on CXR [1,8]. Limitations of LUS regard difficult-to-examine lung areas, particularly the left lung, behind the heart or scapula and central lesions, which are not adjacent to the pleura [2,4]. The time execution of LUS fluctuates from 5 min up to 40–60 min, depending on the child’s collaboration and the pediatrician’s ultrasonography skills [1,5]. Compared with adults, lung ultrasound (LUS) has particular advantages in evaluating the lungs of children due to their thinner chest wall, smaller lung mass and narrower thoracic cavity [11,12,13]. LUS quantifies lung alterations (consolidations, atelectasis, pleural effusion, pulmonary edema, empyema) using measurements and a specific scoring system, based on the absence of air artefacts [13,14,15,16,17]. Different LUS scoring systems have expanded ground since the COVID pandemic because of high accessibility, bedside evaluation and absence of radiation or invasive procedures [17,18]. For children, PedPne LUS score appears to have great precision and an accurate link with inflammatory assessments [18,19,20,21].

The diagnosis of pneumonia in children is assessed using clinical findings (dyspnea, accelerated respiratory rate, respiratory distress, abnormal respiratory sounds), biological findings (inflammatory markers: C-reactive protein (CRP), procalcitonin (PCT), erythrocyte sedimentation rate (ESR), total leukocyte and neutrophil count, gas analysis) and imaging of the lung (LUS and/or CXR) [18,22,23]. Following protocols for standard ill-child evaluation provides good case management, low error rate and successful outcome [13,19,20].

This study aimed to increase the importance of performing LUS in children for CAP diagnosis and monitoring and emphasize its sensitivity, while correlating LUS findings with risk factors, clinical and biological findings, as well as hospital stay prediction.

## 2. Materials and Methods

### 2.1. Study Design

We analyzed in a retrospective study, over an eight-month period (November 2023–June 2024), children admitted and treated for CAP in the Emergency Hospital for Children “Louis Turcanu”, Timisoara.

Inclusion criteria were represented by the following:

- Age between 1 month and 18 years;
- Diagnosis of pneumonia by a specialist doctor;
- Lung ultrasound examination;
- Informed consent signed by a legal guardian.

Exclusion criteria

- Age below 1 month or over 18 years;
- Children admitted with pneumonia but without LUS examination;
- Hospital-acquired pneumonia;
- Lack of informed consent from the patients’ guardians;
- Outpatients with the same diagnosis.

The study was conducted in accordance with the Declaration of Helsinki, and approved by the Ethical Committee of Emergency Hospital for Children “Louis Turcanu”, Timisoara, decision number No. 22/2025, 12 February 2025 and registry number 2357/12 February 2025. Patient confidentiality and privacy were maintained by de-identifying all data before analysis.

### 2.2. Data Collection

Weight was obtained using a standardized scale and was stated in kilograms (kg). Ideal weight was calculated in accordance with World Health Organization (WHO) growth charts, weight-for-age as weight for the 50th percentile [24]. Ponderal index (PI) was calculated by dividing actual weight by ideal weight formula. PI values between 0.9 and 1.1 were considered normal (eutrophic), PI > 1.1—overweight, PI values 0.89–0.76 mild underweight, 0.75–0.61 moderate underweight and <0.6 severe underweight.

Height was obtained using a standardized stadiometer scale and was stated in meters (m). Body mass index (BMI) was calculated using the formula BMI = weight (kg)/height^2^ (m) and was stated in kg/sqm. BMI is recommended for appreciating development in children over 2 years of age [25]. Statistical evaluation of children’s development at the survey moment was performed using the nutritional survey module of the WHO Anthro (for patients under 5 years old) and AnthroPlus (for patients over 5 years old) Survey Analyzer [26,27].

Clinical data was recorded at admission or in the first 24 h and consisted of respiratory rate stated in respirations/minute (rpm) and pulmonary clinical exam. According to patient’s age, normal respiratory rate values were considered: newborn 40–50 rpm, 1–12 months 35–40 rpm, 1–3 years 25–30 rpm, 4–6 years 21–23 rpm, 7–12 years 19–21 rpm and 13–19 years 16–18 rpm [3,28]. Any data above range was considered respiratory distress/polypnea.

### 2.3. Paraclinical Examination

Oxygen saturation was measured with a calibrated pulsoximeter.

Laboratory analysis was performed within the first 24 h after admission and after 3–5 days and consisted of blood gas analysis (PaCO_2_, pH), complete blood count (leucocyte count, neutrophil count, neutrophil percentage (%)) and CRP.

Once parents’ informed consent was obtained, 2 mL of venous blood was collected and placed into EDTA-containing tubes. A total of 1 mL of whole blood sample was used to determine complete blood count on the automated hematology analyzer Sysmex XN1000 (Sysmex Corporation, Kobe, Japan) with impedance spectrometry and flow cytometry. Leucocytes and neutrophil count, and also neutrophil percentage, were interpreted in accordance with normal values for age [29,30].

Blood collected in EDTA tubules was centrifuged at 3000× *g* rotations per minute for 10 min. The resulting plasma was separated. Blood gas analysis used venous blood and was executed on the RapidPoint machine. Normal range values were considered: pH 7.35–7.45; pCO_2_ 41–57 mmHg for venous blood. Biochemical assets: CRP was performed on Cobas Integra 400 Plus by turbidimetry. CRP level was measured in mg/L, and normal range was considered between 0–5 mg/L.

Chest X-ray was accomplished in posterior-anterior incidence and sometimes with lateral incidence, using age-appropriate radiation regimens in the first 24 h after hospital admission, as part of primary investigation protocol for pneumonia and interpreted by a specialist radiologist.

Lung ultrasound (LUS) was performed on each patient in the first 24 h after admission and repeated after 3 to 5 days in selected cases, based on the severity of pneumonia. The examination was performed by the pediatrician using a Philips HD3, version 2.01 ultrasound machine, 7–12 linear MHz probe and 3–5 MHz convex probe for children older than 12 years or with obesity (for better visualization due to the thickness of the adipose panicle). LUS was conducted using a 12-area scoring system [19] that covers six longitudinal sections on each side of the chest as follows: anterior and posterior right and left axillary, right and left mid-clavicular, right and left parasternal, right and left mid-scapular and right and left paravertebral. In addition, the sections were scanned with the probe transversally and evaluated in 3 zones: superior (apical), medial and inferior (basal). Also, we used spleen and liver acoustic windows to examine the costal-diaphragmatic sinuses and the base of the lungs for potential pleural effusion. Most of the patients were examined in the upright position, in their mother’s arms, but for the smaller infants, we used the prone or supine position [19].

We considered potential pneumonia lesion (consolidation), poorly circumscribed hyper-echogenic formations with blurred margins, loss of pleural line and absence of A-lines within the area, with B-line erasing from the deep edge of the consolidations and air bronchograms within the area. LUS can identify minimal consolidation, millimeters in diameter, up to large formations with liver-like appearance [20]. Translobar lesions were to be found in both anterior and posterior examination in the same lobe and with the same length [19]. Pleural effusion was defined as anechoic liquid formation with a sharp border to the pleural wall [8,21]. Every lesion found was scored and noted as follows [6]: more than 3 B-lines per intercostal space—1 point; confluent B-lines—2 points; under 2 cm diameter consolidations—3 points; consolidations > 2 cm diameter (but non-translobar)—4 points; translobar consolidations (lobar pneumonia)—5 points; simple pleural effusion—6 points; complex non-septate pleural effusion—7 points, complex septate pleural effusion—8 points; homogenous hyperechoic effusion (empyema)—9 points [8,19,20,21]. Total (summed) PedPne score was obtained through summation of points for all ultrasonographic findings.

### 2.4. Statistical Analysis

All data were meticulously recorded in a secure computerized database using Microsoft Excel^®^ version 2312 (Build 17126.20132); this version was released on 9 January 2024.

Statistical analysis was conducted using the software package R Version 4.2.3 [31].

Descriptive statistics, such as medians and interquartile ranges (IQRs), were used for numerical variables, whereas frequencies such as percentages (%) and/or counts (n) were used for categorical variables.

Sensitivity was calculated using the following formula: true positive/(false negative + true positive events). To compare the diagnostic performance of LUS and chest X-ray (CXR), we used McNemar’s test for paired binary outcomes with a continuity correction for small numbers of discordant pairs. Because the data set contains only patients with confirmed CAP (n = 102), the test applied to the discordant results in this case looks at LUS true and CXR false cases vs. LUS false and CXR true cases. A significance level of 0.05 was used to assess statistical differences.

The chi-squared test was used to evaluate the association between categorical variables. The paired *t*-test was utilized to compare measurements of clinical scores taken at different time points for the same patients.

The Spearman correlation coefficient (r) was used to assess the strength and direction of the monotonic relationship between two numerical variables. The statistical significance of the correlation was evaluated using a *p*-value.

A Poisson regression model was used to analyze the association between clinical and demographic variables and the length of hospitalization. To prevent overfitting and optimize model parsimony, a stepwise model selection procedure was applied using the Akaike Information Criterion (AIC) [32] as the selection criterion. This process considered both forward and backward directions to identify the combination of predictors that best explained the outcome while minimizing model complexity.

In this study, 95% confidence intervals (CIs) were calculated to provide an estimate of the range within which the true population parameter (such as the mean) is likely to lie. A 95% CI means that if the study were repeated multiple times, 95% of the calculated intervals would contain the true value.

For all statistical tests carried out, the significance level was established as the usual statistical norm of 0.05. However, to control for the increased risk of false positives due to multiple comparisons, a Bonferroni correction [32,33] was applied. The adjusted significance threshold (α = 0.05) is hence divided by the number of tests performed, resulting in an adjusted significance level:

(1) $$\alpha_{\mathrm{adjusted}}=\frac{0.05}{\mathrm{number}\;\mathrm{of}\;\mathrm{hypothesis}\;\mathrm{tests}}$$

Only *p*-values below the more conservative $\alpha_{adjusted}$ threshold were considered statistically significant.

## 3. Results

The study was conducted over an eight-month period (November 2023–June 2024) in the Emergency Hospital for Children “Louis Turcanu”, Timisoara.

**Figure 1:**
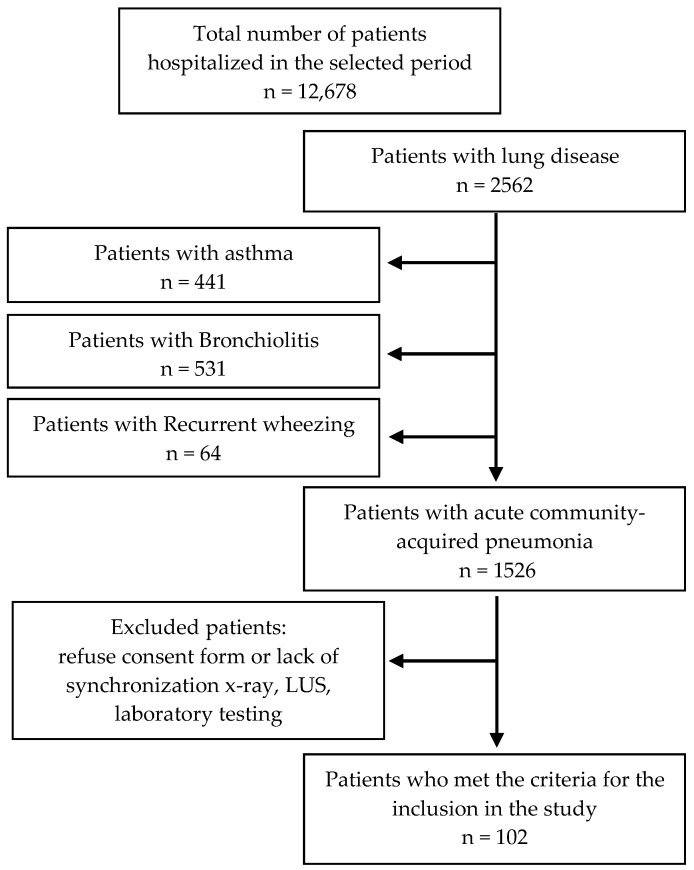
Study population flow chart selection bias.

The hospital is the largest children’s hospital in the western part of Romania, with a high addressability, about 40,000 emergency room presentations/year and 25,000 hospital admissions/year, responsible for up to one fifth of the country’s pediatric population. Within the 8-month study period, the hospital had over 12,000 admissions, while over 2500 had pulmonary involvement (Figure 1).

CAP was responsible for 12.03% of hospital admissions. Inclusion criteria were met by 102 patients. We excluded patients with asthma exacerbations (n = 441), acute bronchiolitis (n = 531) or recurrent wheezing (n = 64), as well as cases in which imagistic and laboratory testing lacked simultaneity, cases with unperformed LUS or lacked of informed consent from legal guardian, thus resulting in a convenience sample of n = 102 patients for this study (Figure 1).

### 3.1. Baseline Characteristics of Study Group

Out of 102 patients included in this study, more than half were males (57.84%), with a male/female (M:F) ratio of 1.37:1. Mean age of patients was 5.7 ± 4.9 years, with a median of 4.2 years. When classifying patients based on age groups, we identified only 14.70% of patients as infants, the majority of patients (49%) being aged 1–6 years of age, congruent with primary collectivity exposure, while just one third of patients (36.27%) were older than 6 years of age. For females, mean age was 5.1 ± 4.3 years, with a median age of 3.7 years, while for male patients, mean age was 6.2 ± 5.2 years, with a median age of 4.4 years (Table 1).

**Figure 2:**
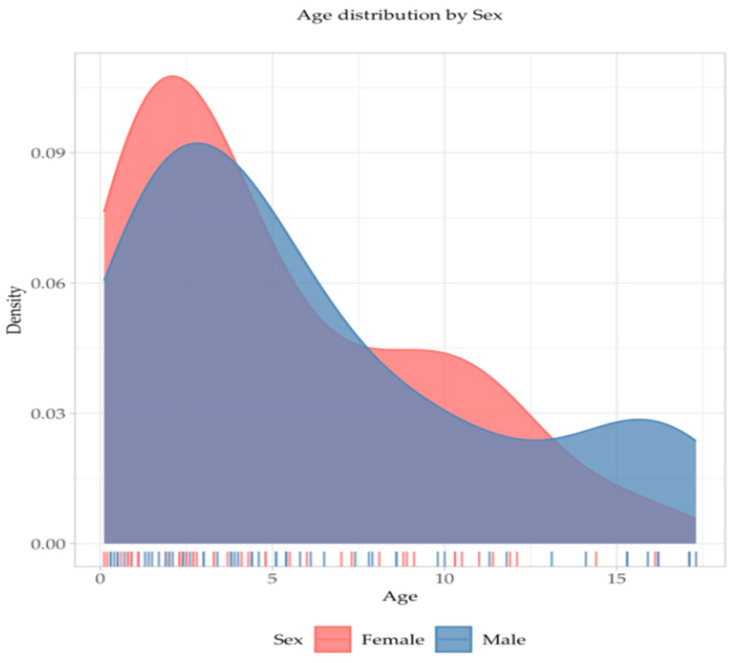
Age distribution by sex in the study group. Red—female patients, blue—male patients.

The majority of patients were young children, aged below 6 (63.72%), with a female predominance in toddlers (Figure 2). Adolescent boys were slightly more frequently affected by CAP than same-age girls.

Environment area was predominantly urban (52.94%), with no statistically significant differences between genders, when compared to a Bonferroni-adjusted significance level of $\alpha_{adjusted}=\frac{0.05}{9}=0.0056$ (Table 2).

### 3.2. Anthropometric Measurements (Weight, Ideal Weight, IP, Height, BMI)

**Figure 3:**
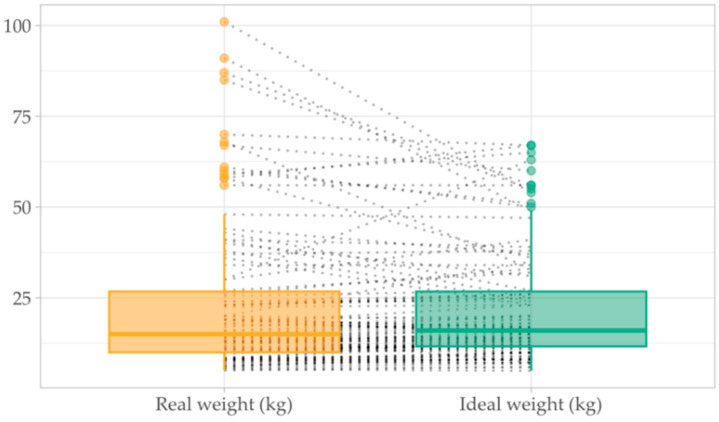
Comparison between the real and ideal weight, based on WHO Child Growth Standards. Mean difference between weight and ideal weight was 1.55 kg (95% CI: −0.47 to 3.57), with a t-statistic of 1.52 and a *p*-value of 0.131.

Weight, height and BMI were interpreted in accordance with age and WHO standards (Figure 3).

We calculated ponderal index (PI) and body mass index (BMI) for female and male patients separately. Analyzing PI values, we determined that 26 patients (25.49%) were overweight, with a preponderance of males (n = 16, 15.68%), and 38 of the patients (37.25%) were underweight for age (Table 3).

Z-score calculation for body mass index (BMI) for age reveals a relation between weight and height (Table 4). In small children, below 5 years of age, almost half (46.55%) were underweight, while in older children, only 40.9% were underweight. Consequently, in older children, overweight was more common (11.36%). Both underweight and overweight in children were considered risk factors for increased pneumonia severity.

### 3.3. Patient-Related Risk Factors for CAP

Medical and environmental history was questioned. Patient-related risk factors for CAP were premature birth, low birth weight, neonatal pathology, chronic diseases, incomplete rickets prophylaxis, smoke exposure, respiratory allergies, collectivity exposure and incomplete vaccination status (Table 2).

Most patients (76.47%) experienced 1–3 risk factors for pneumonia, while twenty-four patients (23.52%) summed up more than four patient-related risk factors as previously described.

### 3.4. Initial Examination

Clinical manifestations for CAP included fever (79.41%), cough (97.05%), difficult breathing, age-related tachypnea (18.62%), sometimes with mild to severe respiratory failure symptoms (20.58%), chest pain (12.74%) or abdominal discomfort in older children, irritability, and poor feeding in smaller infants. Auscultatory pulmonary examinations can vary between normal (34.31%) and crackles or diminished breath sounds (65.68%). Clinical manifestations had a sensitivity in diagnosing pneumonia of 65%.

In the first 24 h after admission, laboratory tests were performed. Blood gas analysis performed revealed pH levels ranging from 7.25 to 8.36, 11 patients with acidosis (pH level < 7.35) and ten patients with alkalosis (pH level > 7.45). None of the patients developed hypercapnia (PaCO_2_ > 57 mmHg).

Complete blood count revealed that only one quarter (25.49%) of patients developed age-correspondent leukocytosis (Table 5), while 31.37% presented with neutrophilia (Table 6). Leucopenia and neutropenia were present in five and six patients, respectively, representing less than 5% of the total number of patients.

CRP ranged between 0.34 mg/L and 338.46 mg/L, with an average value of 45.47 mg/L and a median value of 16.8 mg/L. Elevated CRP (>5 mg/L) levels were identified in 79 patients (77.45%). PCT was determined in 34 patients and ranged between 0.05 ng/mL and 25.33 ng/mL, with an average value of 2.21 ng/mL and a median value of 0.15 ng/mL.

CXR was performed in all patients. Only 41 CXR (40.19%) revealed pulmonary condensations suggestive of acute pneumonia, 55 patients (53.92%) had inflammatory infiltrates, and six patients had no pulmonary X-ray identified modifications. Congruency between CXR and LUS was limited.

**Figure 4:**
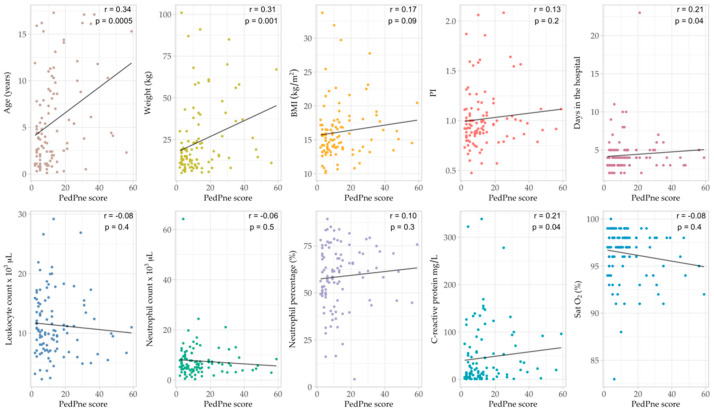
Correlation of summed PedPne score at 24 h with a selection of the variables in the dataset (age, weight, PI, BMI, days in the hospital, leucocyte count at 24 h, neutrophil count at 24 h, neutrophil percentage at 24 h, CRP at 24 h and SatO_2_ at 24 h), Spearman correlation coefficient (r).

LUS was performed in all patients, and scoring system was applied, summing all identified alterations [6,7,8,10]. The total values of LUS score varied between 2 and 59, with a median value of 10. Positive correlations were found between summed PedPne score and age (*p* = 0.0005, r = 0.34), weight (*p* = 0.001, r = 0.31), CRP (*p* = 0.04, r = 0.21) and number of hospitalization days (*p* = 0.04, r = 0.21) (Figure 4). Using a Bonferroni correction to adjust for the 10 hypothesis tests carried out, the significance level is $\alpha_{adjusted}\;=\;\frac{0.05}{10}\;=\;0.005$, meaning that only the correlation between PedPne score and age and PedPne score and weight was found to be statistically significant. Furthermore, the small magnitude of these correlations (r < 0.35) indicates that, although there may be a statistically significant relationship, the clinical utility and predictive power of these variables are limited in this study.

All children included in this study were diagnosed with pneumonia by a specialist pediatrician. Sensitivity for LUS was 100%; all patients presented ultrasound alterations, ranging from confluent B-lines, suggestive of pulmonary inflammatory infiltrates, up to multiple bilateral consolidations and/or complicated pleural effusion. LUS specificity could not be calculated due to a lack of false positive results; this study included only pneumonia-diagnosed patients. Among the 102 patients, chest X-ray (CXR) identified 96 cases and missed six cases (i.e., CXR false while LUS was true). A McNemar test was performed to evaluate whether the difference in detection rates between LUS and CXR was statistically significant. The test yielded a chi-squared statistic of 4.17 (*p* = 0.04), indicating that LUS was significantly more sensitive in detecting CAP compared to CXR. CXR sensitivity was 94%; six patients were diagnosed with CAP and had a negative CXR.

### 3.5. Second Examination

The second evaluation was performed after 72 to 120 h after admission. In only four patients (3.92%), clinical respiratory distress (low oxygen saturation, SaO_2_ < 95%) or tachypnea was still present. Complete blood count, performed in 94 patients (95%), revealed leucopenia in nine patients. Neutropenia was more frequent at the second examination and validated in 30 patients (31.91%), particularly in toddlers. As an imagistic method, LUS was able to be repeated in a short period of time (3 to 5 days) without any unsafe consequences when compared with repeated CXR irradiation dosage. CXR is recommended to be repeated in case of lack of expected improvement, persistent cough, incomplete recovery, recurrent pneumonia or promoted to an advanced imaging technique—Chest Computer Tomography, gold standard for complex lung evaluation.

**Figure 5:**
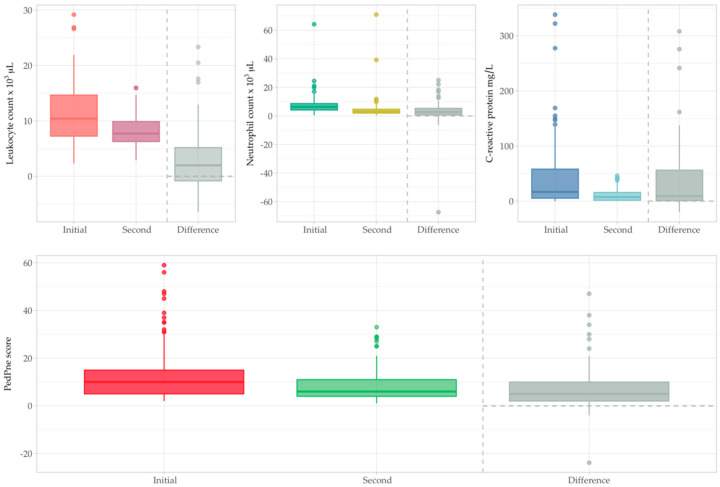
Leucocyte count, neutrophil count, C-reactive protein and summed PedPne comparisons between first and second evaluation.

A paired *t*-test was conducted to compare total leukocyte counts, and the results showed a statistically significant difference (compared with a Bonferroni-adjusted significance level of $\alpha_{adjusted}\;=\;\frac{0.05}{4}\;=\;0.0125)$ between the two time points, with a mean difference of 2.93 × 10^3^/µL (95% CI: 1.81 to 4.04, *p* < 0.001). Neutrophil count paired *t*-test revealed a statistically significant difference, with a mean difference of 2.96 × 10^3^/µL (95% CI: 1.13 to 4.79, *p* = 0.0018) between the two examinations (Figure 5). Both leukocyte and neutrophil counts were significantly higher at 24 h compared to subsequent measurements.

CRP value at second determination ranged between 0.6 mg/L and 46.47 mg/L, with an average value of 10.82 mg/L and a median value of 7.53 mg/L. Elevated CRP was present only in 57 patients (60%). A paired *t*-test comparing C-reactive protein levels at 24 h and at a later time point showed a statistically significant difference, with a mean decrease of 37.33 mg/L (95% CI: 25.08 to 49.58, *p* < 0.001). This reduction in C-reactive protein levels is consistent with the observed trends in leukocyte and neutrophil measurements, supporting the overall resolution of systemic inflammation (Figure 5).

A paired *t*-test comparing the summed PedPne scores at two different time points in 65 patients demonstrated a statistically significant reduction, with a mean difference of 7.76 points (95% CI: 5.12 to 10.39, *p* < 0.001). The significantly lower summed PedPne score at the later time point suggests substantial clinical improvement. This result indicates a marked decrease in the severity of community-acquired pneumonia, reflecting the effectiveness of the clinical management strategies applied in this patient population (Figure 5).

### 3.6. A Predictive Model for Hospital Stay

Hospitalization length varied between 2 and 23 days, with most patients being released after 4 days. A Poisson regression model was used to predict the hospital stay based on several explanatory variables, including the summed PedPne score, age, weight, sex, environmental factors, smoking exposure and several biomarkers (leucocyte count, CRP, neutrophil count and neutrophil percentage). A stepwise model selection procedure based on the Akaike Information Criterion (AIC) was carried out to identify the most parsimonious model that balances goodness of fit with model complexity. The final Poisson regression model includes variables that contribute meaningfully to explaining the number of hospitalization days, as determined by the lowest AIC value among candidate models:

log(E(Days in hospital)) = β_0 + β_1 × Weight + β_2 × Smoker at home + + β_3 × Smoker parents + β_4 × Collective + β_5 × Ponderal Index + β_6 × Environment + β_7 × Antibiotic use at home + β_8 × Neutrophil %

In the Poisson regression model above, the response variable is the number of hospital days. The model uses a log link function to model the expected (average) number of hospital days as a linear function of various clinical and demographic variables. The β coefficients in the equation represent the log-transformed rate ratios for the predictors. The coefficients give insight into how each predictor influences the hospital stay duration, with each coefficient representing the effect on the log-transformed number of days. To interpret the coefficients in terms of the rate ratio, the coefficient can be exponentiated (i.e., e^β), which gives the relative rate of the outcome for each unit increase in the predictor (Table 7).

The Poisson regression model identified several factors associated with the length of hospital stay. Children living in households with smokers had approximately 52% shorter hospital stays compared with those who did not (*p* = 0.01), while parental smoking was associated with a 76% increase in hospital stay duration (*p* = 0.05). Attendance in collective settings was consistently linked to reduced hospitalization times: nursery by 33%, kindergarten by 32%, school by 37% and high school by 51% (all *p* < 0.05). A higher ponderal index (PI) was also significantly associated with a 51% decrease in hospital stay (*p* < 0.01). Other variables, such as weight, neutrophil percentage at 24 h, urban environment and antibiotic use at home, were retained in the model, but their associations with hospital stay were modest and did not reach conventional levels of statistical significance. For instance, each unit increase in weight was associated with an 8% longer stay (*p* = 0.10) and prior antibiotic use with a 22% increase (*p* = 0.06).

## 4. Discussion

### 4.1. Baseline Characteristics of Study Group (Age, Sex, Environment Origin)

The incidence of CAP among children varies based on age, sex, season and health status group. The overall incidence of CAP among children fluctuates between 65.64 and 280 per 1000 person of children presented to health services, with increased incidence rates for developing countries [34]. Based on our patient selection criteria, the incidence of CAP in children admitted to the hospital over the study period was 120.36 per 1000 children. The relatively high incidence in our study can be explained by relating CAP to hospital-admitted children, not to the general population.

In our study group, the majority of patients were aged between 1 and 6 years (49%), in agreement with collectivity exposure, patient mean age was 5.71 ± 4.85 years, and a slightly increased occurrence was noted in male patients (57.84%), M:F 59:43, similar to the literature findings [34,35,36,37,38,39,40].

### 4.2. Anthropometric Measurements (Weight, Ideal Weight, IP, Height, BMI)

Children with malnutrition have a higher risk of developing pneumonia, while pneumonia increases the risk of malnutrition [41]. Analyzing PI and z-scores for body weight index, this study reported that underweight children had a prevalence of 44.11%, higher in children aged below 5 years. Prevalence of underweight children diagnosed with pneumonia is generally high and varies between studies and countries, ranging between 19.6 and 66.3%, with a median of 40.2%, indicating a link between poverty-related factors and pneumonia [41,42]. On the other hand, other studies reported that overweight or obese children are more prone to develop CAP due to the excess adipose tissue, which secondarily produces inflammatory mediators, thus contributing to increased inflammation and triggering an immune response in the lungs [43].

### 4.3. Patient-Related Risk Factors for Pneumonia

Incomplete vaccination and malnutrition have been quoted as risk factors for pneumonia in children less than five years [44]. Other factors like low birth weight, premature birth, pathological prenatal conditions, chronic diseases, inadequate Vitamin A intake, low Zinc intake, summary parental education, low socioeconomic status, none exclusive breastfeeding, indoor pollution (smoke exposure within the household), reduced access to medical care, distance to water source, use of wood as fuel source, caring of a child on mother while cooking and overcrowding within the household are also known to increase the risk of CAP development and increased severity [37,44,45,46,47]. In our study, we analyzed presence of premature birth (22.54%), low birth weight (12.74%), respiratory distress at birth (4.9%), at least one other lower respiratory tract infection (44.11%), incomplete rickets prophylaxis (41.17%), indoor pollution/smoke exposure (48.03%), respiratory/alimentary allergies (20.58%), collectivity-exposure (70.59%) and incomplete immunization (40.19%) as risk factor for pneumonia in children.

### 4.4. Initial Examination (Clinical, Inflammatory Markers and Imaging)

Systematic clinical examination with abnormal/modified breath sounds and tachypnea is highly evocative of acute pneumonia. Most existing studies define pneumonia based on clinical signs, while others consider pulmonary involvement only when confirmed by imaging techniques [48,49,50,51]. In the present study, diagnosis of pneumonia was both clinical and radiologically confirmed; also, lung ultrasound was performed in every case at least once.

Clinical aspect of a child with pneumonia includes fever (79.41%), cough (97.05%), difficult breathing, age-according tachypnea (18.62%), and sometimes with mild to severe respiratory failure symptoms (20.58%), similar with literature data, although some features vary depending on the study group population, as chest pain is mainly encountered in children older than 5 years while poor feeding is mostly developed by toddlers [48,49,50,51,52]. Auscultatory pulmonary examinations vary from normal breath sounds (21.56%) to the presence of crackles or diminished breath sounds (76.47%). Clinical auscultation is user-dependent and cannot be objectively quantified, although, when comparing, other studies mention an incidence of pulmonary crackles of 42.9% [52]. These different results may be explained by different clinical protocols and chest X-ray recommendations. Rare pneumonia clinical manifestations include reduced consciousness, convulsions, pallor and headache [48,49,50,51,52].

Difficulty in breathing, tachypnea, nasal flaring and grunting are clinical signs for respiratory distress and, along with hypoxia, may lead, in severe cases, to hypercapnia. In our study, none of the patients had developed hypercapnia. Current WHO guidelines for children under 5 years of age distinguish simple cough from pneumonia based on the presence/absence of tachypnea [52]. Pulse oximetry is used frequently to assess the severity of respiratory distress, while levels below 95% require oxygen administration. In our study, 25.49% of patients needed oxygen administration.

Pneumonia can be caused by bacteria, viruses or fungi. In order to diagnose etiology differently, microbiological cultures are needed, but microbiological testing is challenging to obtain or is very expensive. More frequently, CAP viral etiology is oriented according to clinical findings: high fever, wheezing or rapid onset of symptoms, along with negative inflammatory tests, leucopenia and lymphocytosis [53,54]. Bacterial CAP is characterized by crackles or asymmetric breath sounds, slow onset of symptoms, prolonged fever, increased inflammatory markers, elevated CRP and PCT, leukocytosis and neutrophilia [53,54,55]. Presence of leukocytosis in childhood pneumonia indicates an increased grade of inflammation (60–70%) in comparison with tuberculosis or viral infections [55,56]. In our study, leukocytosis was encountered in 25.49% according to age-adapted normal values, while neutrophilia was identified in 34.31%, and only a few cases (less than 5%) showed leucopenia or neutropenia. Increased white blood count shows a sensitivity ranging from 40 to 83% and a specificity of 49 to 100% [57]. Using a combined increased white blood count along with increased CRP marker can raise sensitivity and specificity up to 100%, while CRP alone is known to have a sensitivity of 64% and a specificity of 63% [57]. PCT markers have considerably higher accuracy than do CRP markers for discerning bacterial infections from non-infectious causes of inflammation, in a meta-analysis, the pooled sensitivity for PCT markers was 88% (95% CI, 80–93%), compared with 75% (95% CI, 62–84%) for CRP marker [57,58]. Several studies indicate a cut-off value of 50–65 mg/L for viral vs. bacterial infection [58]. In the present study, mean CRP value was 16.8 mg/L, and more than 78% of patients presented CRP above 5 mg/L, while only 30.39% had CRP values above 50 mg/L.

Imaging examinations in pneumonia include chest X-ray, LUS and thoracic CT. Currently, chest X-ray is the most common imaging technique for pediatric pneumonia. Although accessible, chest X-ray implies a low level of radiation and has a reduced specificity for smaller-than-1 cm pulmonary consolidations [5,9,10,59]. Sensitivity of CXR varies between 90–92% with a specificity of 87–93%, a negative predictive value of 88.5% for pneumonia diagnosis [5,60]. Once, with the emergence of the COVID-19 pandemic, there was an increased interest in performing LUS as a radiation-free, inexpensive, portable and easy diagnosis tool, with a bedside approach, less-radiant and repeatable at any time [60,61]. Sensitivity of LUS varies between 95 and 100%, with a specificity of 97–100% and a negative predictive value of 100% for consolidation recognition [5,60]. Chest CT remains the most sensitive imaging technique for pulmonary evaluation, although less accessible and with greater costs, longer duration and increased radiation exposure [60]. Radio-occult pneumonia diagnosed by LUS, unseen by CXR, can be encountered in 9–15% patients, depending on the localization of pneumonia (retrocardiac area), consolidation dimensions and LUS operator’s experience [5,8,60,61,62]. In our study, radiological occult pneumonia was encountered in six patients (5.88%), while for more than half of the patients (53.92%), CXR revealed inflammatory infiltrates. These inflammatory infiltrates may be visible as a thickened or hazy area on CXR and usually indicate an area of the lung where inflammation occurred, caused by viral, bacterial or fungal infection or other conditions. The difference between inflammation and thickening cannot always be discerned on the CXR [63,64]. CXR consolidations were found in 40.19% of patients. LUS scoring system is often used to appreciate the entanglement of pneumonia. Summing all LUS-identified alterations is a valid option [19,20,21,65]. In the present study, summed PedPne score varied between 2 and 59, with a median value of 10; the higher values are explained by miscellaneous etiology of CAP and summing-lesion-PedPne algorithm [19]. LUS has a high specificity (100%), and its findings may properly appreciate the extent of lung alterations. LUS can easily monitor the evolution of CAP, increasing the chances of early identification of potential complications. Also, LUS does not imply radiation and can be performed in multiple conditions, also at the patient’s bedside, reducing the administrative investigation delay. CXR has shown a sensitivity of only 94% when identifying any modification of the lung, while in detecting consolidations, sensitivity lowers to 44.82%. Thoracic CT is very sensitive, but it is expensive, requires an increased dose of irradiation, is time-consuming and sometimes requires anesthesia for the patient. Performing LUS in the proximity of the patient, the bedside approach can improve early CAP diagnosis, increase comfort and reduce unnecessary delay and radiation exposure.

The Spearman correlation reveals a statistically significant but weak positive correlation between the summarized PedPne score and age (*p* = 0.0005, r = 0.34) and weight (*p* = 0.001, r = 0.31). Although some variables showed statistically significant correlations with the PedPne score, the magnitude of these associations was weak (r < 0.35), suggesting limited predictive or clinical utility. This underscores the importance of interpreting statistical findings within a clinical context. The aggregation of LUS findings into the summed PedPne score did not yield significant improvements when correlating with inflammatory markers as compared to previous studies [5,8,19,20,65].

### 4.5. Second Examination

Second examination included clinical, biological and ultrasound PedPne score and exposed progress of CAP under-treatment. Favorable evolution was encountered in 101 patients; only one patient died due to comorbidities and respiratory failure. Biologically, neutropenia was more frequently encountered (31.91%) after 3–5 days of treatment, especially in toddlers. A substantial decline in leucocyte count (*p* < 0.001), neutrophil count (*p* = 0.0018), C-reactive protein (*p* < 0.001) and PedPne score (*p* < 0.001) was demonstrated between the two evaluation points.

### 4.6. A Predictive Model for Hospital Stay

General recommendations for the treatment course for CAP in children range from 5 to 10 days [66]. WHO recommends a 3- to 5-day course of oral Amoxicillin for immunocompetent children with pneumonia in order to reduce antibiotic overuse and increased resistance [67]. National Romanian guidelines recommend a 7- to 10-day course of antibiotics for non-complicated CAP in children [3]. Hospitalization is recommended for moderate to severe CAP, particularly in the presence of risk factors or complications [63].

In our study, hospitalization varied between 2 and 23 days, with a median value of 4 days. The hospital-stay Poisson regression model was based on weight, environment, exposure to household smokers, collective attendance, ponderal index, antibiotic use at home and neutrophil percentage at 24 h revealing that attending school (higher age) or normal nutritional status generally leads to a shorter hospital stay, while having parents who smoke or living in an urban environment might increase the stay.

We are aware of the study limitations: Limitations of LUS regard difficult-to-examine lung areas, particularly the left lung, behind the heart or scapula and central lesions, that are not adjacent to the pleura. The study cohort is rather small and selective, as were included only children with CAP who were admitted to the hospital. A key limitation of this study is that LUS examinations were performed and interpreted by a single physician without blinding or formal reliability assessment. Although this ensured consistency, it limits generalizability and introduces the potential for subjective bias in scoring. Future studies should include multiple operators and assess interrater reliability to validate findings. Another limitation of this study resides in not including a control group, resulting in the inability to calculate LUS and CXR specificity and predictive value.

The strength of the present study lies in being the first to employ a comprehensive summed PedPne score that consolidates all pathological LUS findings. This study correlates these findings with clinical, biological and imagistic features in children with CAP, therefore highlighting the utility and sensitivity of LUS in pediatric assessment.

Whether LUS can be correlated with CAP’s severity or sepsis risk remains debatable, and further studies need to analyze this specific topic. LUS findings complete the diagnosis of CAP in children, rather than being able to predict etiology, treatment or CAP severity; other factors must be identified, and additional research is needed.

## 5. Conclusions

Diagnosing CAP can often be straightforward, particularly when key clinical aspects are present, such as abnormal/modified breath sounds, fever and cough. However, when clinical and radiological findings are lacking or inconclusive, LUS is a sensitive and highly specific method for diagnosing and monitoring CAP in children. Moreover, LUS is a measurable and reproducible method that not only offers a less stressful experience for the pediatric patient but is also extremely financially feasible, with a sensitivity of 100%; therefore, it is mandatory to consider including LUS in the diagnosis algorithm of CAP.

## Figures and Tables

**Table 1:** Age–sex distribution of patients. n—number, SD—standard deviation, IQR—interquartile range.

Sex	n	%	Min Age	Max Age	Mean Age	Median Age	SD Age	IQR Age
Female	43	42.2%	0.1	16.1	5.1	3.7	4.3	6.9
Male	59	57.8%	0.3	17.3	6.2	4.4	5.2	7.1
Total	102	100%	0.1	17.3	5.7	4.2	4.9	6.8

**Table 2:** Demographic data in the study group.

	All n = 102	Feminine n = 43	Masculine n = 59	*p* Value *
Age				0.53
<1 year	15	8 (18.60%)	7 (11.86%)	
≥1 years, <3 years	25	12 (27.90%)	13 (22.03%)	
≥3 years, <6 years	25	8 (18.60%)	17 (28.81%)	
≥ 6 years	37	15 (34.88%)	22 (37.28%)	
Environment origin				0.48
Urban	54	25 (58.13%)	29 (49.15%)	
Rural	48	18 (41.86%)	30 (50.84%)	
Medical History				
Premature birth (gestational age ≤ 37 weeks)	23	9 (20.93%)	14 (23.72%)	0.30
Small weight at birth (birth weight ≤ 2500 g)	14	5 (11.62%)	9 (15.52%)	0.28
At least one episode of pneumonia	45	19 (44.18%)	26 (44.06%)	0.29
Incomplete Rickets prophylaxis	34	19 (44.18%)	15 (25.42%)	0.49
Smoke exposure at home	48	22 (51.16%)	26 (44.06%)	0.56
Allergy	17	6 (13.95%)	11 (18.64%)	0.22

* Chi-square test.

**Table 3:** Sex-related distribution of ponderal index (PI) and nutritional status.

Sex	n	Mean PI	SD PI	CI	Severe Underweight	Moderate Underweight	Mild Underweight	Eutrophic	Overweight
Female	43	1.00	0.27	0.91–1.08	3 (2.94%)	2 (1.96%)	11 (10.78%)	17 (16.66%)	10 (9.8%)
Male	59	1.03	0.31	0.95–1.12	1 (0.98%)	6 (5.88%)	15 (14.70%)	21 (20.58%)	16 (15.68%)

PI—ponderal index, SD—standard deviation, CI—confidence interval, n—number.

**Table 4:** Z-score calculation for BMI for age—WHO Child Growth Standards.

	Z-Score (Value)	Percentile (%)	<5 Years n = 58 (%)	>5 Years n = 48 (%)
Severe underweight for age	<−3 SD	<0.1%	3 (5.17%)	6 (13.63%)
Mild and moderate underweight for age	from −1 SD to −3 SD	2.3–15.9%	24 (41.37%)	12 (27.27%)
Normal for age	from −1 SD to 1 SD	15.9–84.1%	26 (44.82%)	18 (40.9%)
Possible risk of being overweight	>1 SD	>84.1%	3 (5.17%)	3 (6.81%)
Overweight for age	>2 SD	>97.7%	2 (3.44%)	5 (11.36%)

WHO—World Health Organization, SD—standard deviation, n—number.

**Table 5:** White blood cell normality [26] and study findings.

Age/ Patients in Age Group (n)	Normal Leucocyte Count (×10^3^/mmc)	Leucocyte Range in Study Group (×10^3^/mmc)	Leucopenia in Study Group n (%)	Leukocytosis in Study Group n (%)
1 month—1 year (n = 15)	6–17.5	6.7–20.6	1 (6.66%)	4 (26.66%)
1–12 years (n = 75)	5–14.5	2.3–29.17	5 (6.66%)	21 (28%)
13–18 years (n = 12)	4.5–14.5	4.5–15.5	1 (8.33%)	1 (8.33%)

**Table 6:** Absolute neutrophil count (ANC), neutrophils (%) in accordance with age groups [26] and alterations (neutropenia, neutrophilia).

Age/ Patients in Age Group (n)	Absolute Neutrophil Normal Count (×10^3^/mmc)	Neutrophil Normal Range (%)	Neutropenia in Study Group n (%)	Neutrophilia in Study Group n (%)
1 month–1 year (n = 15)	1–9	20–48	-	5 (33.33%)
1–6 years (n = 51)	1.5–8	37–71	6 (11.74%)	15 (29.41%)
7–18 years (n = 36)	1.8–8	33–76	-	13 (36.11%)

**Table 7:** Poisson regression model coefficients.

Variable	Estimate	Std. Error	z-Value	*p*-Value
Intercept	1.86	0.30	6.17	<0.001 *
Weight	0.01	0.01	1.63	0.10
Smoker at Home	−0.74	0.29	−2.54	0.01 *
Smoker Parents	0.57	0.29	1.94	0.05
Nursery (vs. home)	−0.40	0.17	−2.42	0.02 *
Kindergarten (vs. home)	−0.38	0.15	−2.61	0.01 *
School (vs. home)	−0.46	0.18	−2.56	0.01 *
High School (vs. home)	−0.68	0.33	−2.03	0.04 *
Ponderal Index	−0.72	0.23	−3.17	<0.01 *
Urban Environment (vs. Rural)	0.19	0.10	1.85	0.06
Antibiotic Use at Home	0.20	0.10	1.88	0.06
Neutrophil % (24 h)	0.01	0.00	1.46	0.15

A positive coefficient indicates that as the predictor variable increases, the expected log count of hospital days increases, meaning the patient is expected to stay longer in the hospital. A negative coefficient suggests that as the predictor variable increases, the expected log count of hospital days decreases, meaning the patient is expected to stay for a shorter duration. * statistically significant *p* value *p* < 0.05. Intercept: Exponential Estimate (exp(1.86) ≈ 6.42): The baseline expected number of days in the hospital is approximately 5.42 days when all other predictors are at their reference levels.

## Data Availability

The information is contained within this article. For additional information, please feel free to inquire with either the original author or the corresponding author. The public’s access to the data is restricted as a result of the patient privacy standards that regulate the handling of clinical data.
